# Evaluating auditory stream segregation of SAM tone sequences by subjective and objective psychoacoustical tasks, and brain activity

**DOI:** 10.3389/fnins.2014.00119

**Published:** 2014-06-06

**Authors:** Lena-Vanessa Dolležal, André Brechmann, Georg M. Klump, Susann Deike

**Affiliations:** ^1^Animal Physiology and Behavior Group, Department for Neuroscience, School for Medicine and Health Sciences, Center of Excellence “Hearing4all,” Carl von Ossietzky University OldenburgOldenburg, Germany; ^2^Special Lab Non-invasive Brain Imaging, Leibniz Institute for NeurobiologyMagdeburg, Germany

**Keywords:** auditory scene analysis, amplitude modulation, temporal and spectral cues, time shift detection, BOLD response, fMRI

## Abstract

Auditory stream segregation refers to a segregated percept of signal streams with different acoustic features. Different approaches have been pursued in studies of stream segregation. In psychoacoustics, stream segregation has mostly been investigated with a subjective task asking the subjects to report their percept. Few studies have applied an objective task in which stream segregation is evaluated indirectly by determining thresholds for a percept that depends on whether auditory streams are segregated or not. Furthermore, both perceptual measures and physiological measures of brain activity have been employed but only little is known about their relation. How the results from different tasks and measures are related is evaluated in the present study using examples relying on the ABA- stimulation paradigm that apply the same stimuli. We presented A and B signals that were sinusoidally amplitude modulated (SAM) tones providing purely temporal, spectral or both types of cues to evaluate perceptual stream segregation and its physiological correlate. Which types of cues are most prominent was determined by the choice of carrier and modulation frequencies (*f*_mod_) of the signals. In the subjective task subjects reported their percept and in the objective task we measured their sensitivity for detecting time-shifts of B signals in an ABA- sequence. As a further measure of processes underlying stream segregation we employed functional magnetic resonance imaging (fMRI). SAM tone parameters were chosen to evoke an integrated (1-stream), a segregated (2-stream), or an ambiguous percept by adjusting the *f*_mod_ difference between A and B tones (Δ*f*_mod_). The results of both psychoacoustical tasks are significantly correlated. BOLD responses in fMRI depend on Δ*f*_mod_ between A and B SAM tones. The effect of Δ*f*_mod_, however, differs between auditory cortex and frontal regions suggesting differences in representation related to the degree of perceptual ambiguity of the sequences.

## Introduction

In everyday life, the auditory system organizes acoustic signals based on similarities and differences in their sound features (Bregman, 1990). Especially in complex acoustic scenes, spectral or temporal stimulus parameters affect the grouping and segregation processes in assigning sounds to different sources. Sounds from one source are perceived as one coherent auditory stream. Studies of auditory stream segregation commonly applied the ABA- paradigm (e.g., Van Noorden, 1975; Moore and Gockel, 2002, 2012). The amount of stream segregation depends on the differences in sound features, i.e., the physical differences between A and B signals. It has been proposed that these differences will lead to the representation of the signals assigned to the separate streams by separate populations of neurons being differentially activated in time (e.g., Fishman et al., 2001; Elhilali et al., 2009). Furthermore, the separate representation of the A and B signals can be observed already at the first stages of auditory processing (i.e., the cochlear nucleus) as Pressnitzer et al., 2008 demonstrated. Previous studies evaluated the auditory streaming percept and the neural mechanisms underlying the perceptual organization of sounds elicited by spectral (e.g., Van Noorden, 1975; Fishman et al., 2001, 2004; Bee and Klump, 2004, 2005; Deike et al., 2004, 2010; Micheyl et al., 2005; Micheyl and Oxenham, 2010) and temporal differences (Grimault et al., 2002; Vliegen and Oxenham, 1999; Roberts et al., 2002; Gutschalk et al., 2007; Itatani and Klump, 2011; Dolležal et al., 2012b) between A and B signals. These studies have provided evidence how auditory streaming is affected by a variety of simple features. Although applying a common paradigm, the outcome of these studies may also depend on the psychoacoustical task that was employed or on the measure that was used for evaluating the segregation of the streams. The majority of the psychoacoustical tasks relied on a subjective perceptual judgment (e.g., Van Noorden, 1975; Vliegen and Oxenham, 1999; Grimault et al., 2002; Roberts et al., 2002; Micheyl et al., 2005; Gutschalk et al., 2007; Micheyl and Oxenham, 2010; Dolležal et al., 2012b). In the subjective task, subjects simply report their streaming percept. Few studies employed objective tasks (Van Noorden, 1975; Neff et al., 1982; Vliegen et al., 1999; Cusack and Roberts, 2000; Roberts et al., 2002; Micheyl and Oxenham, 2010; Thompson et al., 2011). In the objective task, the subject's perceptual threshold is determined using stimulus conditions in which threshold sensitivity is enhanced by one perceptual organization and hampered by the other (i.e., 1- and the 2-stream percept). Thus, in the objective task the streaming percept is inferred from the measured perceptual sensitivity. The different measures that were used range from the evaluation of perception to the assessment of the brain activity by applying invasive or non-invasive measurement techniques. Since few studies compared results obtained with different tasks (Micheyl and Oxenham, 2010) and measures (Gutschalk et al., 2007; Wilson et al., 2007), we have only little evidence how well these results are correlated.

Here, we investigate the correlation of the extent of stream segregation for sinusoidally amplitude modulated (SAM) A and B signals across two different psychoacoustical tasks and across two different measures (i.e., subjective psychoacoustical task and fMRI) providing a comprehensive approach to auditory stream segregation. The comparison across different psychoacoustical tasks involves an objective and a subjective task presenting signals with identical sound features to the same subjects. We propose that the thresholds obtained in the objective task are correlated with the subjective percept of stream segregation indicating that either task allows measuring the amount of perceptual stream segregation. Since any salient difference between sequential signals may elicit stream segregation (Moore and Gockel, 2002, 2012), we expect that a correlation will be found irrespective whether temporal or spectral cues can be utilized to differentiate between A and B signals. As is outlined in the methods below, SAM signals offer temporal, spectral, or both types of cues for stream segregation dependent on the modulation frequency (*f*_mod_) and carrier frequency (*f_c_*), respectively. By an appropriate choice of *f_c_* and *f*_mod_ for the SAM tone stimulus sequences the amount of stream segregation between A and B SAM tones elicited by spectral cues and temporal cues can be varied (Dolležal et al., 2012a).

The comparison across measures includes the combination of the subjective task with fMRI. Previous human fMRI and MEG studies using either spectral (Deike et al., 2004, 2010; Gutschalk et al., 2005; Snyder et al., 2006; Wilson et al., 2007) or temporal (Gutschalk et al., 2007) differences between A and B stimuli consistently showed an increase of activity throughout the auditory cortex combined with a change of the dominant percept from 1-stream to 2-stream with increasing difference between stimuli. Based on these results we propose that A and B SAM tones show the same Δ*f*_mod_ dependent activity in auditory cortex irrespective of the type of cue (i.e. spectral vs. temporal). The second goal pursued in obtaining fMRI activity measurements was to find further evidence of the specific involvement of regions outside the auditory cortex in stream segregation which is still an open question in auditory streaming research. Cusack (2005), e.g., found that the intraparietal sulcus was differentially involved depending on the perceptual organization of physically identical stimuli in perceptual ambiguous sequences with a stronger BOLD activation for the segregated two-stream compared to the integrated one-stream percept. Such a segregation specific activation in the absence of physical differences was not found by Dykstra et al. (2011) when using intracranial EEG in neurosurgical patients with epilepsy. However, he observed a Δf dependent activity in middle temporal gyrus, pre- and post-central gyri, inferior and middle frontal gyri, and the supra-marginal gyrus. The human fMRI study by Kondo and Kashino (2009) found neural correlates of perceptual switching in the posterior insula, thalamus, and supra-marginal gyrus. Finally, some other studies did not describe evidence for the involvement of regions outside auditory areas in stream segregation (e.g., Wilson et al., 2007).

## Materials and methods

### Subjects

#### Psychoacoustical measurements

Six human subjects (age 25–44 years, mean age 30 years, five females, including the first author) participated in two main experiments (Experiment 1 and 2; ABA- sequences) comparing subjective and objective psychoacoustical measures of stream segregation. All subjects had normal audiograms, with absolute pure tone thresholds <20 dB hearing level in the range from 0.25–10 kHz. Four of the subjects had previous experience with psychoacoustic experiments. In a control experiment (applying the conditions of Experiment 1 in B-only sequences) four of the subjects (age 25–42 years, mean age 30 years, three females, including the first author) participated. All experiments were undertaken with the understanding and written informed consent of each subject, following the Code of Ethics of the World Medical Association (Declaration of Helsinki). The experiments were approved by the local ethics committee of the University of Oldenburg. In addition to these psychoacoustical measurements in quiet, the subjective streaming percept was determined during fMRI (see fMRI measurements).

#### fMRI measurements

In Experiment 1, 13 subjects (age 20–31 years, mean age 26 years, five female) and in Experiment 2, 10 subjects (age 20–33 years, mean age 26 years, four female) participated. One subject participated in both experiments. Due to technical problems, psychophysical data of one subject in Experiment 2 are missing and only the data of 9 subjects were analyzed. All but one subject were right-handed (Edinburgh Handedness Inventory; laterality quotient ≥ +45) and this one subject was ambidextrous (laterality quotient: 18). All showed a language laterality toward the left hemisphere tested as described in Bethmann et al. (2007). The subjects gave written informed consent to the study that was approved by the Ethics Committee of the University of Magdeburg. Five additional participants were excluded from the final analysis: one because of more than five missing responses and four because of head movements during the fMRI-measurement that were stronger than 2.3 mm translation and/or 2.3° rotation.

### Apparatus, stimuli, and procedure

#### Stimuli

In the present study ABA- sequences (the dash indicates a silent interval of the same duration as the signal duration) were presented that consisted of fully sinusoidally amplitude modulated tones (SAM). ABA- sequences are commonly applied to determine the amount of stream segregation by varying the physical difference between A and B signals (Van Noorden, 1975). A and B signals with small physical differences are perceptually grouped into a single sequence (i.e., 1-stream percept) with a galloping rhythm (i.e., ABA-ABA-ABA-…), whereas A and B signals with large physical differences are perceptually segregated to two streams (i.e., 2-stream percept) with different isochronous rhythms (i.e., A-A-A-A-A-A-… and -B---B---B--…). For A and B signals with intermediate physical differences subjects may have an ambiguous percept, that is characterized by a switching between the 1- and the 2-stream percept (e.g., Moore and Gockel, 2002, 2012).

The SAM tones were digitally synthesized in Matlab (Version 7.1) at a sampling frequency of 44.1 kHz and produced by a Hammerfall DSP (Multiface II, RME). These signals (10 ms raised cosine rise/fall) had a duration of 125 ms and were presented at an overall presentation level of 70 dB SPL with a tone repetition time (TRT) of 250 ms. SAM tones have the advantage that the carrier frequency (*f_c_*) and the modulation frequency (*f*_mod_) can be adjusted in such a way that, depending on the parameter values and the auditory filter bandwidth (Kohlrausch et al., 2000), they provide either temporal, spectral, or both types of cues for stream segregation (Dolležal et al., 2012a). Dolležal et al. (2012a) used a computational model of the auditory periphery to calculate excitation pattern differences of A and B SAM tones and estimate spectral stream segregation thresholds based on these differences. If the observed thresholds were below the prediction based on spectral cues alone (i.e., could not be explained by spectral cues), they concluded that only temporal cues were relevant for the segregated percept. If the observed thresholds were similar or higher than the thresholds predicted on the basis of spectral cues, it was concluded spectral cues could provide a basis for perceptual stream segregation (for more details see Dolležal et al., 2012a). Table 1 summarizes the different parameter settings and highlights conditions in which spectral cues alone could explain stream segregation. For the remaining parameter settings, spectral cues are unlikely to explain stream segregation.

**Table 1:** **Stimulus conditions for Experiment 1 and Experiment 2**.

***f_c_* [kHz]**	***f*_mod A_ [Hz]**	**Small Δ*f*_mod_[Hz]**	**Small Δ*f*_mod_[oct]**	**Medium Δ*f*_mod_ [Hz]**	**Medium Δ*f*_mod_[oct]**	**Large Δ*f*_mod_ [Hz]**	**Large Δ*f*_mod_[oct]**
**EXPERIMENT 1, EFFECTS OF *f*_mod A_**							
1	100	33	0.415	100	1.0	300	2.0
1	300	29	0.135	100	0.415	300	1.0
**EXPERIMENT 2, EFFECTS OF *f*_c_**							
1	100	33	0.415	100	1.0	300	2.0
4	100	33	0.415	100	1.0	300	2.0

*For each experiment, the carrier frequency (f_c_), the modulation frequency of the A SAM tone (f_mod A_) and the modulation frequency difference between A and B SAM tones (*Δ*f_mod_) are indicated.* Δ*f_mod_ conditions that are printed in bold typeface show conditions in which stream segregation was can be explained on the basis of spectral cues (Dolležal et al., 2012a) estimated from a model calculating excitation pattern differences between the A and the B SAM tones in the inner ear. Spectral cues provided by* Δ*f_mod_ conditions that are printed in regular typeface are unlikely to be sufficient for stream segregation.*

Here, ABA- sequences consisted of SAM tones that had the same carrier frequency (*f_c_*) but different modulation frequencies (*f*_mod_). Note the *f*_mod_ of the A SAM tones (*f*_mod A_) was always lower than the *f*_mod_ of the B SAM tones (*f*_mod B_). For the psychoacoustical tasks and the fMRI measurements, in the two different experiments the effect of *f*_mod A_ (Experiment 1) or the effect of the *f_c_* (Experiment 2) on stream segregation was analyzed. In Experiment 1 SAM tones had an *f_c_* of 1 kHz and an *f*_mod A_ of either 100 or of 300 Hz and in Experiment 2 SAM tones had an *f_c_* of either 1 or of 4 kHz and an *f*_mod A_ of 100 Hz. For each condition three *f*_mod_ differences between A and B SAM tones (Δ*f*_mod_) were chosen to evoke a 1-stream, a 2-stream and an ambiguous percept for the tested conditions, respectively (see Table 1). The value of Δ*f*_mod_ was adjusted based on the study by Dolležal et al. (2012a). Dolležal et al. (2012a) also presented ABA SAM tone sequences, but they used either a TRT of 125 ms or a TRT of 375 ms for SAM tones of 125 ms duration. Based on their results the preset study chose for both experiments Δ*f*_mod_ stimulus conditions that enable a comparison of stream segregation elicited by spectral and non-spectral cues. In Experiment 1 such a comparison can be made at the medium Δ*f*_mod_ condition and in Experiment 2 at the large Δ*f*_mod_ condition (Table 1; see Dolležal et al., 2012a). The results obtained in the present study were compared across two different psychoacoustical tasks and across two different measures (i.e., subjective psychoacoustical task and fMRI) for all Δ*f*_mod_ stimulus conditions.

#### Psychoacoustical measurements

Psychoacoustical data were obtained in two different locations (Oldenburg and Magdeburg). In Oldenburg all subjects participated in the subjective and in the objective task in quiet in a sound-attenuating chamber (IAC, Industrial Acoustics Company, Mini 250). The stimuli were presented diotically with calibrated headphones (Sennheiser HDA 200). In Magdeburg, subjects participated in the subjective task during fMRI. Written instructions and additional verbal explanations, if necessary, were given to the subjects before the beginning of the tasks.

**Figure 1:**
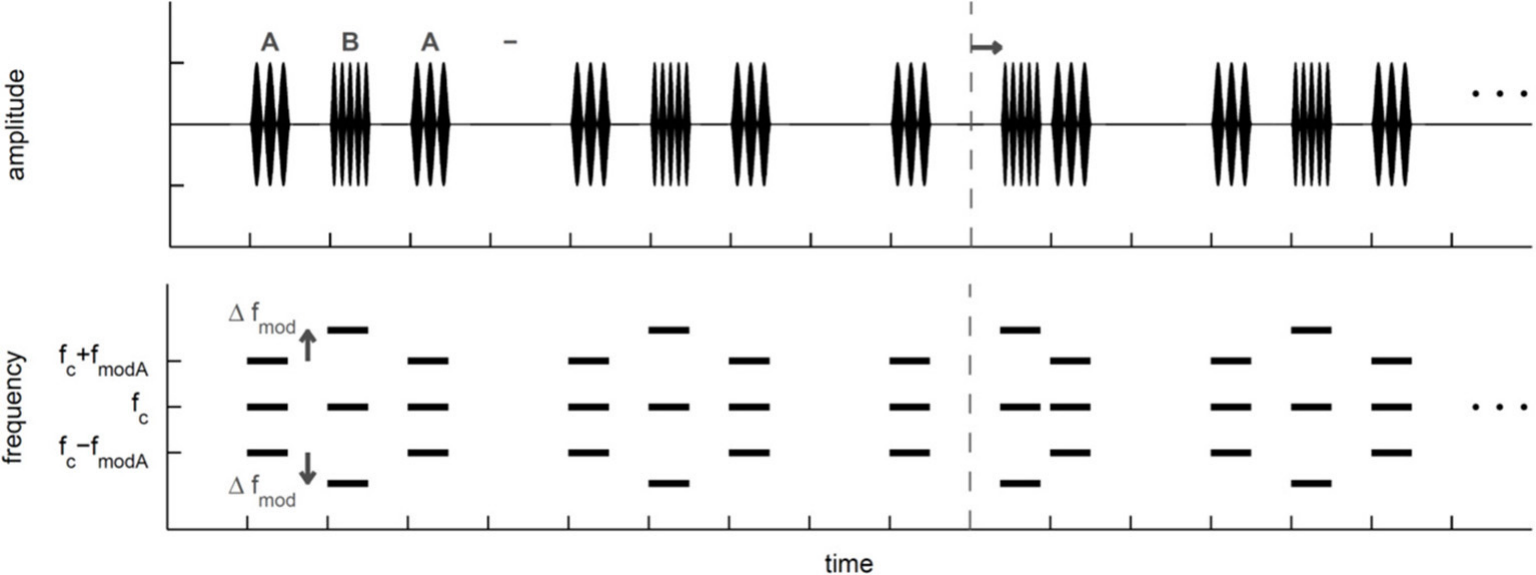
**Schematic view of the ABA-triplets presented in the objective task that relied on the detection of a time shifted B signal.** In the third ABA- triplet a black arrow indicates the shift of the B signal, whereas the dashed line indicates the former position the un-shifted B signal. Top: Schematic temporal view of the ABA- triplets that were sinusoidally amplitude modulated (SAM) tones. A and B SAM tones had the same carrier frequency (*f_c_*) but different modulation frequencies (*f*_mod_). The *f*_mod_ of the B SAM tone was always larger than the *f*_mod_ of the A SAM tone (*f*_mod A_). Here the *f*_mod_ difference between A and B SAM tones (Δ*f*_mod_) is schematically shown (see Table 1 for exact values). Bottom: Schematic spectral view of the SAM ABA- triplets.

**Figure 2:**
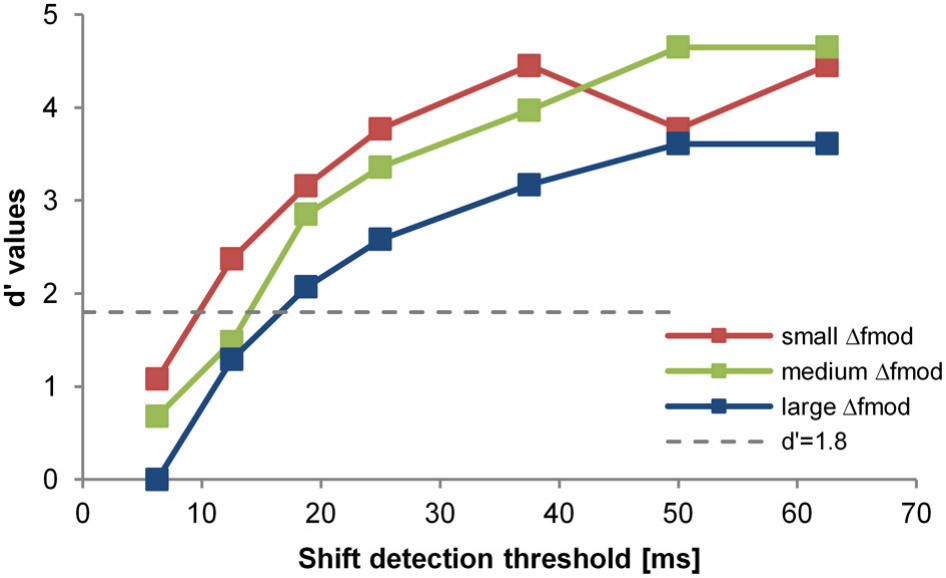
**Psychometric function of one subject for one stimulus condition (i.e., exp. 2, *f_c_* = 4 kHz).** The *d*'-value is plotted in relation to the shift of the B-signal in ms (x-axes). The differently colored lines and symbols show the different Δ*f*_mod_ conditions tested (see legend).The threshold criterion of *d*' = 1.8 is indicated by the dotted gray line. The shift detection threshold (*d*' = 1.8) was interpolated between data points lying above and below that *d*'-value. The slight differences in largest *d*' values are due to different false alarm rates for the different Δ*f*_mod_ conditions.

***Objective task in quiet***. Subjects started the experiment with the objective task in quiet. To measure objectively the perceptual segregation of the A and B SAM tones, subjects performed a shift detection task (Figure 1) in a Go/NoGo experiment determining the detection of a time shifted B SAM tone in the ABA- sequence. Thresholds obtained with the shift detection task should be smaller for ABA- sequences that are perceptually integrated into one stream than for ABA- sequences that are perceptually segregated to two streams (e.g., Van Noorden, 1975). In the present study, subjects listened to the presentation of a repeated ABA- triplet without a time shifted B SAM tone. Within 1 to 7 s (randomized time interval) after subjects started a trial by pressing a button on the touch screen either a forward shifted B SAM tone (Go-stimulus) replaced the regular B SAM tone or no replacement took place and a regular B SAM tone was presented (NoGo-stimulus, 30% of trials). If subjects detected the Go-stimulus in time (response latency < 1 s) by pushing a button on a touch screen, a correct response (hit) was registered and a green light flashed. If the subjects missed the Go-stimulus, a miss was recorded and the next trial was automatically initiated. The Go-response in this complex time-shift detection task could be based on the evaluation of the time interval between the A SAM tone and the successive B SAM tone or on the time interval between two sequential B SAM tones. Responses to NoGo-stimuli (false alarms) were registered too. Hit and false alarm rates were used to calculate the sensitivity measure d' (Green and Swets, 1966; see data analysis, psychoacoustics). For threshold estimation in each stimulus condition (Table 1) subjects had to complete a minimum of three sessions consisting of one obligatory training session and two subsequent test sessions (within each session a specific Go-stimulus was presented 10 times). A session lasted for about 20 min and consisted of eleven blocks of ten trials each. The first block of each session served as a warm-up block in which only the most salient Go-stimuli were presented. Each of the remaining ten blocks consisted of seven different Go-stimuli and three NoGo-stimuli that were presented in a random order. The Go-stimuli with a time shifted B SAM tone (step size 6.25 or 12.5 ms; i.e., 5 or 10% of the SAM tone duration) were chosen according to the method of constant stimuli. The range of the time shifts imposed on the B SAM tone was individually adjusted before each session to provide both sub-threshold and supra-threshold Go-stimuli. After each session a psychometric function was constructed relating the hits and misses of seven different Go-stimuli (different amounts of a time shifted B SAM tone) to *d*'-values (a measure of sensitivity for detecting the shift; see Figure 2). Between threshold sessions presenting different stimulus conditions a minimum pause of 5 min occurred. Within the objective task in quiet, the threshold estimation for the different stimulus conditions was randomized.

In addition to the objective task in quiet presenting ABA- sequences with time shifted B SAM tones, a control experiment (for all stimulus conditions of Experiment 1) was conducted presenting B-only sequences. In B-only sequences only B SAM tones (omitting the A SAM tones) were presented (-B---B---B--) resulting in a TRT of 1000 ms. This experiment mimics a condition with a completely segregated percept, in which subjects solely rely on the stream of B SAM tones for the shift detection.

***Subjective task in quiet***. After performing in the objective task in quiet, subjects participated in the subjective task in quiet. Here, the same stimulus conditions as in the objective task were applied. ABA- sequences (15 s duration) of each stimulus condition were presented six times in randomized order. A pause of 45 s was introduced between the presentation of ABA- sequences of different *f_c_* and *f*_mod A_. After the presentation of each ABA- sequence subjects were instructed to indicate their percept (e.g., 1- or 2-stream percept) on a touch screen (Elo, 1542L, 15”, Rear-Mount Touch-monitor). Then, the next ABA- sequence with another randomly chosen stimulus condition was initiated. Before starting the experiments in the subjective task in quiet, subjects attended a training session to familiarize with the task.

***Subjective task during fMRI***. During fMRI measurements, the subjects were presented with the same stimuli as in the subjective task in quiet. The duration of the stimulus sequences was increased to 16 s to adapt to the repetition time (*TR* = 2000 ms) of the functional echo planar imaging (EPI) sequence. Each of the three conditions (small, medium and large Δ*f*_mod_) were presented 10 times for each *f*_mod A_ (100, 300 Hz) in Experiment 1 and for each *f_c_* (1, 4 kHz) in Experiment 2, respectively, resulting in the presentation of 60 sequences per experiment. For each experiment, the order of the 60 sequences was pseudo-randomized with silence blocks of 16 s duration in between, which served as baseline condition. The stimuli were presented diotically via fMRI compatible headphones (Baumgart et al., 1998) at an individually adjusted, comfortable sound level, using Presentation (Neurobehavioral Systems Inc., San Francisco, USA). During the fMRI measurements, the subjects' heads were fixed with a cushion with attached earmuffs containing the headphones. Additionally, the subjects wore earplugs.

Prior to the fMRI measurements, the subjects received written instructions and additional verbal explanations if necessary. The subjects were asked to listen to the sound sequences and to indicate their percept at the end of each sequence by pressing the left button on a response panel with their right index finger when they perceived the SAM tones as one coherent stream, and the right button with their right middle finger when they perceived them as two separate streams. All button presses were recorded using Presentation (Neurobehavioral Systems Inc., San Francisco, USA) to test the perception of the SAM tone sequences under background scanner noise conditions. To familiarize the subjects with the sound sequences and the task, prior to the actual measurements, they were exposed to sequences, which most likely promote one or the other perceptual alternative, i.e., the 1-stream and the 2-stream percept, respectively.

#### fMRI measurements and data acquisition

The study was carried out on a 3 Tesla scanner (Siemens Trio; Erlangen, Germany) equipped with an eight channel head coil. A three-dimensional anatomical data set of the subject's brain (192 slices of 1 mm each) was obtained before the functional measurement. Additionally, before each functional run an Inversion-Recovery-Echo-Planar-Imaging (IR-EPI) with the identical geometry as in the functional measurement was acquired. Functional volumes were collected using a continuous EPI sequence (echo time *TE* = 30 ms; *TR* = 2000 ms; flip angle = 80°; 32 slices; matrix size = 64 × 64; field of view (FOV) = 19.2 cm^2^, 3 mm isotropic resolution). The total experiment comprised 968 volumes scanned in 32 min 16 s.

### Data analysis

#### Psychoacoustical measurements

Psychoacoustical data were analyzed with repeated-measures analyses of variance (rmANOVAs, IBM SPSS Statistics Version 21.0). In all rmANOVAs, we report the *F*-values, the *p*-values and the partial η^2^, a non-additive value representing the “variance accounted-for” measure of the effect size, which can vary from 0 to 1 for the main effects. *Post-hoc* Tukey tests were Bonferroni corrected.

***Objective task***. For the threshold estimation of a stimulus condition data from two consecutive valid sessions in which thresholds differed by no more than 6.25 ms (i.e., 5% of the SAM tone duration) from each other were combined. A session was accepted as being valid based on two criteria: (1) Subject had a mean hit rate of 80% of the two easiest Go-stimuli (largest time shifts of the B SAM tone) and (2) their false alarm rate (NoGo-stimuli) was below 20%. Based on the rates of hits and misses, a psychometric function was constructed relating *d*'-values to each of the time shifts. By linearly interpolating between adjacent values of the psychometric function a shift detection threshold was determined as the time shift resulting in a *d*'-value of 1.8 (Green and Swets, 1966: Figure 2). To exclude training effects, the stimulus conditions of each experiment were randomized. Furthermore, after thresholds for all stimulus conditions were obtained subjects had to repeat the threshold measurement for the first condition of the series. If the new shift detection threshold differed by more than 6.25 ms from the shift detection threshold obtained in the first run subjects had to repeat measurements until the new threshold matched the threshold obtained in the first measurement (threshold difference ≤ 6 ms). In these cases the repeated shift detection threshold was taken for further analysis, discarding the previously measured threshold. In the rmANOVA, the shift detection thresholds were analyzed in relation to the stimulus condition (Δ*f*_mod_) and *f*_mod A_ (Experiment 1) or *f_c_* (Experiment 2).

***Subjective task***. For each subject and each condition the mean proportion of a 2-stream percept was calculated from the presentations of 6 (in quiet) or 10 sequences (during fMRI), respectively, per condition and then averaged across subjects. The proportion of a 2-stream percept in relation to the stimulus condition (Δ*f*_mod_) and *f*_mod A_ (Experiment 1) or *f_c_* (Experiment 2) was analyzed in a rmANOVA. The effect of the condition of presentation (in quiet or during fMRI) on the proportion of a 2-stream percept was tested as between-subjects factor.

#### fMRI measurements

The functional data were analyzed using BrainVoyager™ QX (Brain Innovation, Maastricht, Netherlands). A standard sequence of pre-processing steps, such as 3D-motion correction, linear trend removal, and filtering with a high-pass of three cycles per scan was performed. The functional data sets were projected to the IR-EPI-images, co-registered with the 3D-data sets, and then transformed to Talairach space.

For each experiment separately, a conjunction analysis using a multi-subject random-effects general linear model (RFX-GLM) was performed to identify brain regions which showed positive deflections of the BOLD signal in at least one of the 3 conditions compared to the baseline (*t* ≥ 4.5, *p* < 0.002 (uncorrected for multiple comparisons), cluster threshold: 108 mm^3^) for each of the two stimulus variants:

Experiment 1: *f*_mod A_ 100 Hz > baseline AND *f*_mod A_ 300 Hz > baseline,
Experiment 2: *f_c_* 1 kHz > baseline AND *f_c_* 4 kHz > baseline.

The analysis included %-transformed functional data of all subjects and used the standard 2-gamma hemodynamic response function implemented in BrainVoyager™ QX. From the resulting clusters volumes-of-interest (VOIs) were defined. The BOLD responses of each VOI were subjected to repeated-measures analyses of variance (rmANOVAs) testing for the within factors condition (Experiment 1 and 2: small, medium and large Δ*f*_mod_), *f*_mod A_-variant (Experiment 1: 100, 300 Hz) and *f_c_*-variant (Experiment 2: 1, 4 kHz). *Post-hoc* pair wise comparisons were performed using RFX-GLM analyses.

## Results

### Psychoacoustical measurements in quiet and during fMRI

The perceptual segregation of SAM tones was evaluated using either the subjective task (subjects directly reported their perceptual state in quiet or during fMRI) or the objective task that relied on the detection of a forward shifted B SAM tone within the ABA- sequence. In the first experiment the effect of *f*_mod A_ was evaluated, whereas in the second experiment the effect of *f_c_* was evaluated. Both, a variation of *f*_mod A_ as well as a variation of *f_c_* affects the representation of the SAM tones by temporal and/or spectral cues.

### Experiment 1—the effect of the modulation frequency of the a SAM tone (*f*_mod A_)

#### Subjective task

**Figure 3:**
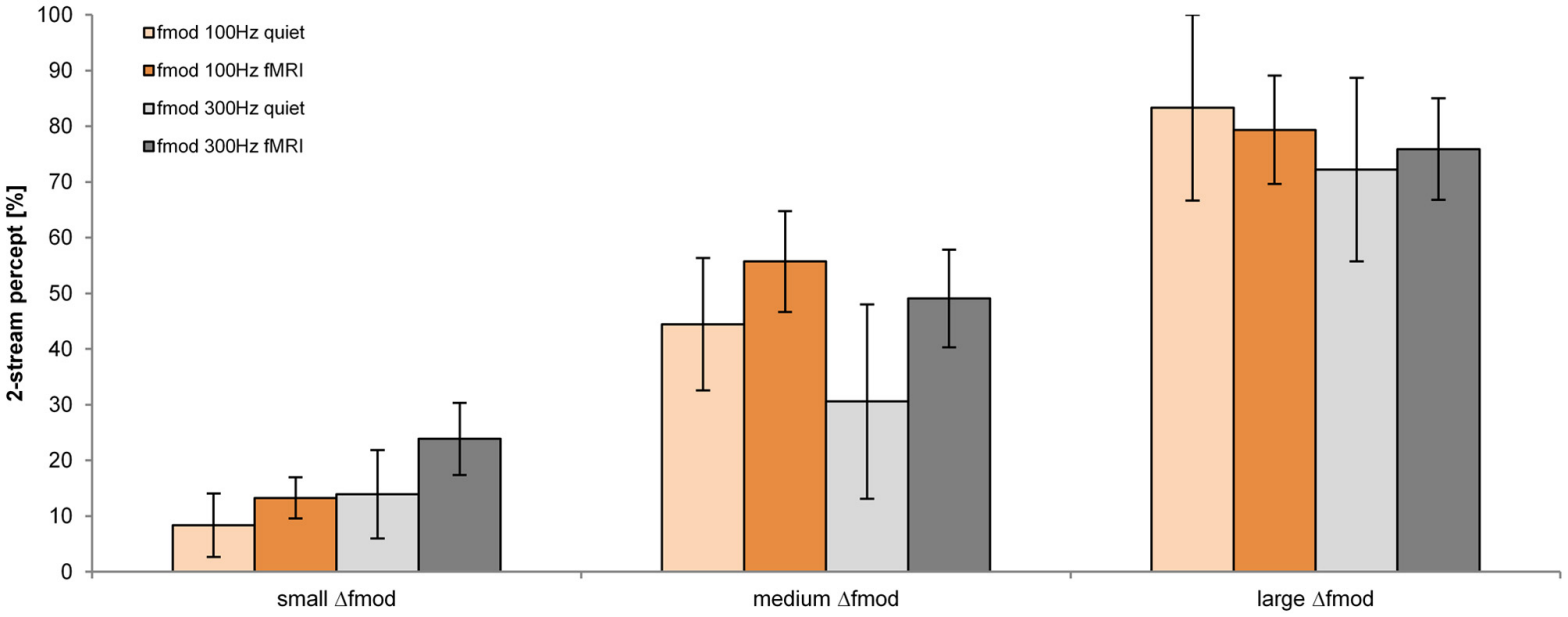
**Proportions of a 2-stream percept (mean and s.e.m.) are shown for the *f*_mod A_ of 100 Hz (orange) and 300 Hz (gray) for the measurements in quiet (lighter coloring: *n* = 6) and during fMRI (darker coloring: *n* = 13) for all Δ*f*_mod_ conditions**.

The proportion of a 2-stream percept depended significantly on the stimulus condition Δ*f*_mod_ [*F*_(2, 34)_ = 31.755; *p* < 0.001, η^2^ = 0.651]. The *f*_mod A_ and the condition of presentation (in quiet and during fMRI) did not have a significant effect on the proportion of a 2-stream percept (Figure 3). Pair-wise comparisons showed a significant difference in the proportion of a 2-stream percept between all tested Δ*f*_mod_ stimulus conditions (all *p* ≤ 0.003). The mean proportion of a 2-stream percept increased significantly with increasing Δ*f*_mod_ condition showing the least mean proportion of a 2-stream percept of 16.2% for ABA- sequences presented with the small Δ*f*_mod_ condition. For ABA- sequences presented with the medium Δ*f*_mod_ condition a mean proportion of a 2-stream percept of 47.7% was observed. The largest mean proportion of a 2-stream percept of 77.7% was observed for the large Δ*f*_mod_ condition. No significant interaction was found.

#### Objective task in quiet

**Figure 4:**
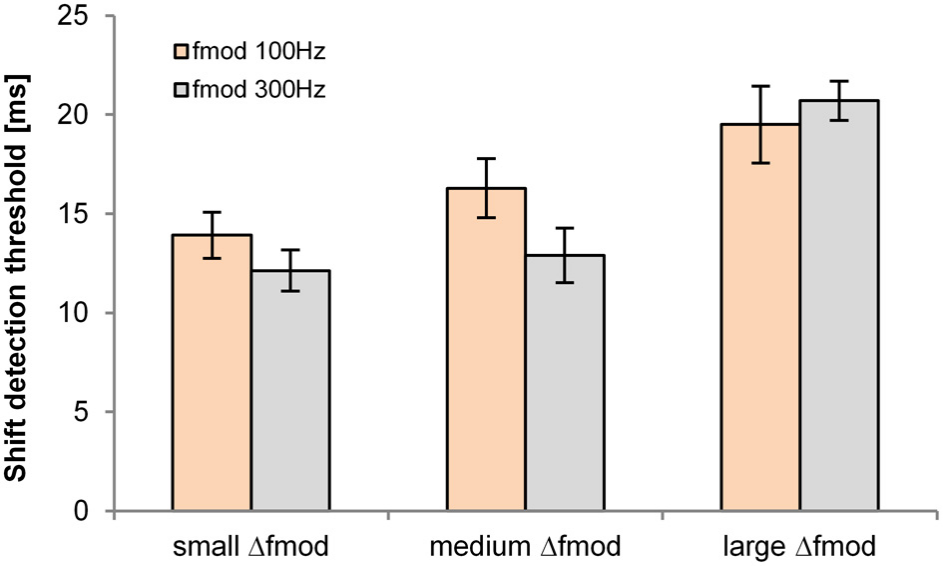
**Shift detection thresholds of the B SAM tone (*n* = 6; mean and SEM) are shown for the Δ*f*_mod A_ of 100 Hz (orange) and 300 Hz (gray) for all Δ*f*_mod_ conditions**.

The shift detection threshold of the B signal was significantly affected by the Δ*f*_mod_ stimulus condition [*F*_(2, 10)_ = 38.795; *p* < 0.001, η^2^ = 0.886, Figure 4]. No significant main effect of *f*_mod A_ on the shift detection threshold was observed. Pair-wise comparisons showed significantly higher shift detection threshold for the large (mean = 20.1 ms) than for the small (mean = 13 ms; *p* = 0.001) and medium Δ*f*_mod_ condition (mean = 14.6 ms; *p* = 0.001). No significant difference between the shift detection threshold of the small and the medium Δ*f*_mod_ condition was observed and no significant interaction was found.

Whether B SAM tones were presented by themselves (control experiment presentation of B-only sequences) or together with A SAM tones (only large Δ*f*_mod_ condition of the main experiment, presentation of ABA- sequences) had a significant effect on the shift detection thresholds [*F*_(1, 3)_ = 34.272; *p* = 0.01, η^2^ = 0.920]. Pair-wise comparisons showed significant higher mean shift detection thresholds for the control experiment (48.3 ± 2.4 ms) than observed in the large Δ*f*_mod_ condition of the main experiment (mean = 20.1 ± 1.1 ms).

### fMRI measurements

Table 2 lists all brain regions which were commonly activated or deactivated (*t* = 4.5, *p* < 0.002), respectively, by both *f_modA_* in at least one of the three conditions (small, medium, and large Δ*f*_mod_) compared to the baseline condition.

**Table 2:**
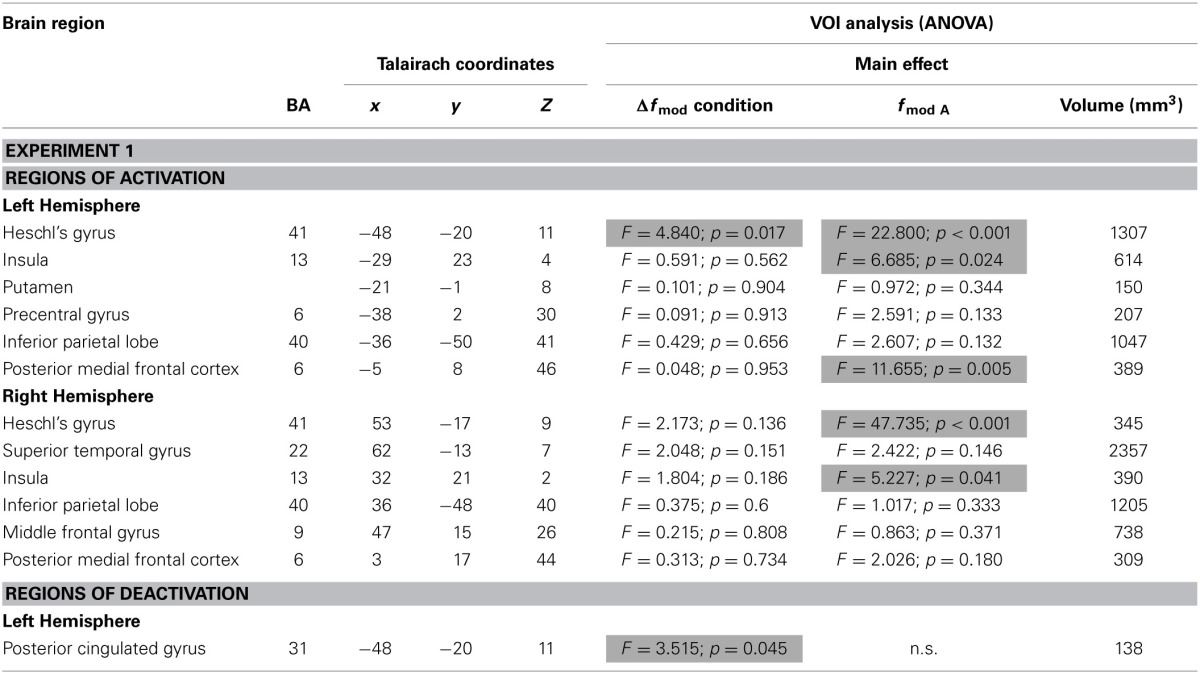
**Brain regions (BA-Brodmann area; *x,y,z*-Talairach coordinates) showing positive or negative deflections of the BOLD signal in at least one of the three Δ*f*_mod_ conditions compared to the baseline (*t* ≥ 4.5, *p* < 0.002) for each of the two *f*_mod A_ (100, 300 Hz) tested in Experiment 1 and the results of ANOVAs within the resulting VOIs**.

Significant effects are highlighted in gray.

**Figure 5:**
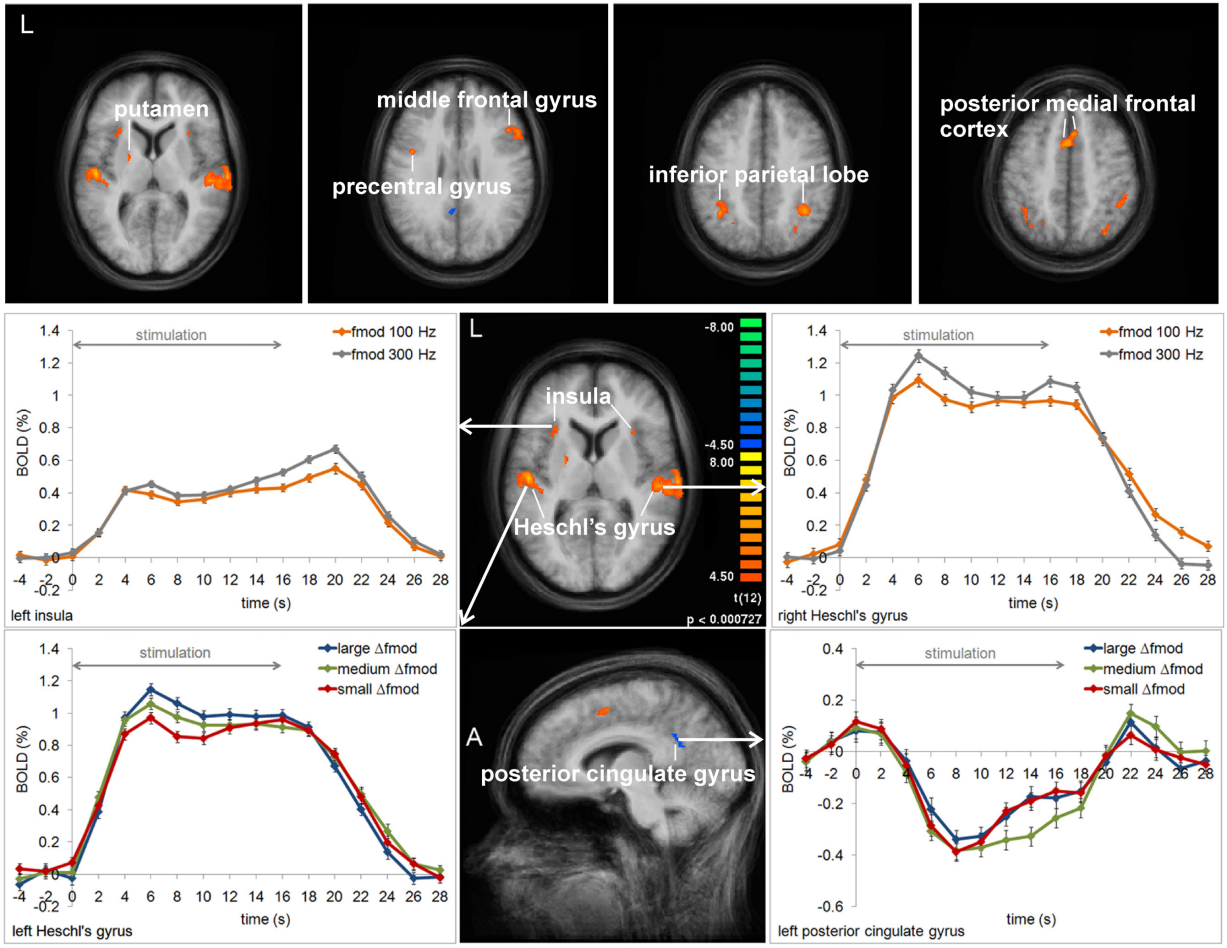
**Group average activation maps (13 subjects) and BOLD signal time courses within regions of interest in Experiment 1.** The maps depict all brain regions showing positive or negative deflections of the BOLD signal in at least one of the three Δ*f*_mod_ conditions compared to the baseline (*t* = 4.5, *p* < 0.002) for each of the two *f*_mod A_ (100, 300 Hz). Several regions that showed significant differences between conditions and *f*_mod A_ variants are labeled and the respective averaged BOLD signal time courses are assigned. Error bars represent SEM.

In Experiment 1, the ANOVAs of BOLD responses within the respective VOIs revealed a main effect of Δ*f*_mod_ condition in left Heschl's gyrus (HG) [F_(2, 24)_ = 4.840, *p* = 0.017] and left posterior cingulated gyrus (PCG) [F_(2, 24)_ = 3.515, *p* = 0.045]. In the left HG the BOLD response amplitude increased with increasing Δ*f*_mod_ (see Figure 5), The *post-hoc* tests showed a significant difference between the small and the large Δ*f*_mod_ condition (*t* = 2.892, *p* = 0.013) and a trend between the medium and the large Δ*f*_mod_ condition (*t* = 2.102, *p* = 0.057). In left PCG, the *post-hoc* tests showed a significantly stronger negative deflection of the BOLD signal of the medium compared to the small Δ*f*_mod_ condition (*t* = 3.465, *p* = 0.005).

In addition, in left and right HG [*F*_(1, 12)_ = 22.800, *p* < 0.001; *F*_(1, 12)_ = 47.735, *p* < 0.001], left and right insula [*F*_(1, 12)_ = 6.685, *p* = 0.024; *F*_(1, 12)_ = 5.227, *p* = 0.041], and the left posterior medial frontal cortex (pMFC) [*F*_(1, 12)_ = 11.655, *p* = 0.005] a main effect of *f*_mod A_ was found with higher BOLD response amplitudes during *f*_mod A_ 300 Hz compared to *f*_mod A_ 100 Hz stimulation (see Figure 5). There was no significant interaction of the factors Δ*f*_mod_ condition and *f*_mod A_.

### Experiment 2—the effect of the carrier frequency (*f_c_*)

#### Subjective task

**Figure 6:**
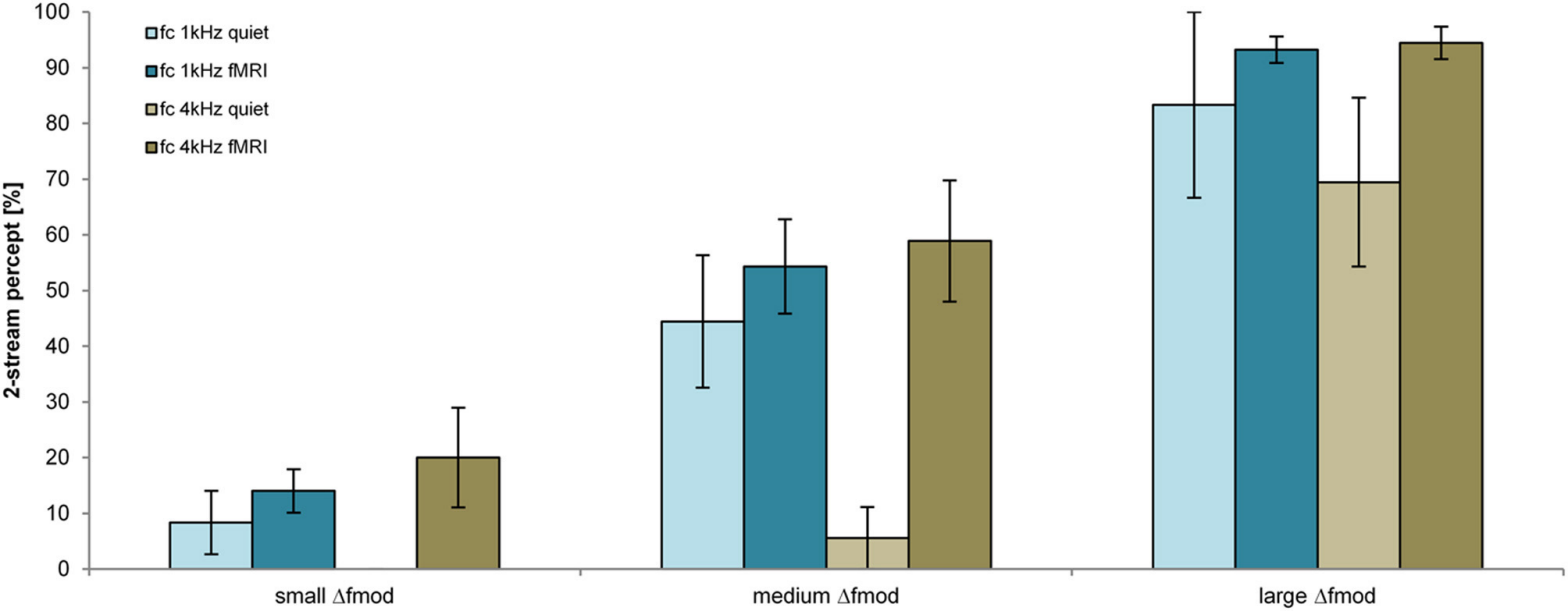
**Proportions of a 2-stream percept (mean and s.e.m.) are shown for the *f_c_* of 1 kHz (blue) and 4 kHz (brown) for the measurements in quiet (lighter coloring: *n* = 6) and during fMRI (darker coloring: *n* = 9) for all Δ*f*_mod_ conditions**.

The proportion of a 2-stream percept depended significantly on the Δ*f*_mod_ condition [*F*_(2, 26)_ = 51.595; *p* < 0.001, η^2^ = 0.799], on the *f_c_* of the SAM tones [*F*_(1, 13)_ = 11.623; *p* = 0.005, η^2^ = 0.472] and on the condition of presentation [in quiet or during fMRI; *F*_(1, 13)_ = 8.168; *p* = 0.013, η = 0.386; Figure 6]. Pair-wise comparisons showed a significant difference in the proportion of a 2-stream percept between all tested Δ*f*_mod_ conditions (all *p* ≤ 0.009). The proportion of a 2-stream percept increased significantly with increasing Δ*f*_mod_ (mean percentage of a 2-stream percept for the small Δ*f*_mod_ = 11.9%, medium Δ*f*_mod_ = 44.0% and large Δ*f*_mod_ = 86.9%). ABA- SAM tone sequences presented with the lower *f_c_* of 1 kHz showed a significantly higher proportion of a 2-stream percept (50.5%) than SAM tones of the higher *f_c_* of 4 kHz (44.7%). The proportion of a 2-stream percept measured in quiet was significantly smaller (mean = 35.2%) than the proportion of a 2-stream percept measured during fMRI (mean = 55.8%). The Two-Way interaction of the factors *f_c_* and condition of presentation was significant (*p* < 0.001), showing a significant higher proportion of a 2-stream percept for the lower *f_c_* of 1 kHz in quiet (mean = 45.4%) than for the higher *f_c_* of 4 kHz in quiet (mean = 25.0%; *p* = 0.006), whereas the proportion of a 2-stream percept during fMRI was not affected by the *f_c_*. No other interaction was significant.

#### Objective task in quiet

**Figure 7:**
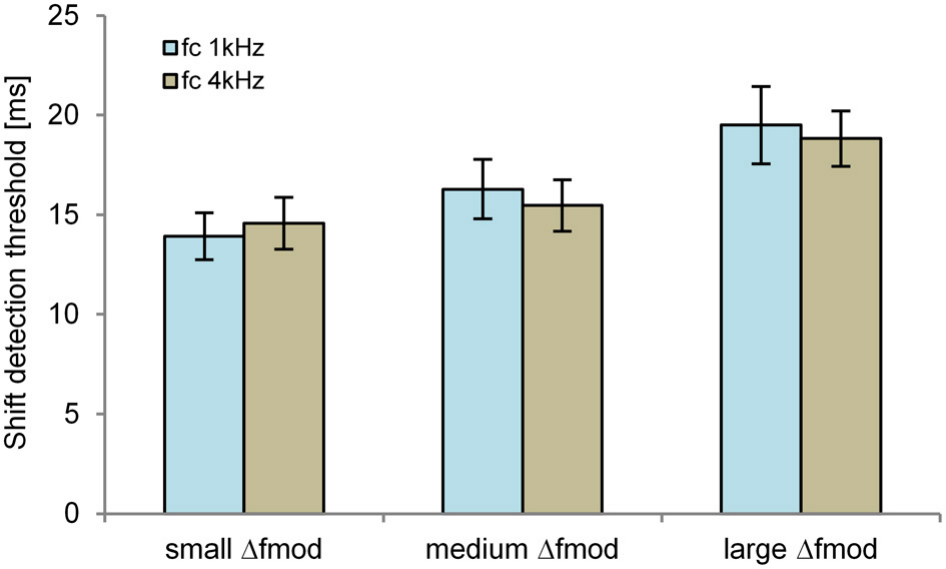
**Shift detection thresholds of the B SAM tone (*n* = 6; mean and SEM) are shown for the *f_c_* of 1 kHz (blue) and 4 kHz (brown) for all Δ*f*_mod_ conditions**.

The detection threshold of the time shifted B SAM tone of the ABA- sequence was significantly dependent on the stimulus condition Δ*f*_mod_ [*F*_(2, 10)_ = 10.018; *p* = 0.004, η^2^ = 0.667, Figure 7]. No significant main effect of *f_c_* on the shift detection threshold was observed. Pair-wise comparisons showed a significantly smaller shift detection threshold for the small (mean = 14.2 ms) than for the large Δ*f*_mod_ stimulus condition (mean = 19.2 ms; *p* = 0.01). No significant difference between the shift detection threshold of the medium (mean = 15.9 ms) and the small and large Δ*f*_mod_ was observed. No significant interaction was found.

### fMRI measurements

Table 3 lists all brain regions which were commonly activated (*t* = 4.5, *p* < 0.002) by both *f_c_* in at least one of the three conditions (small, medium, and large Δ*f*_mod_) compared to the baseline condition.

**Table 3:**
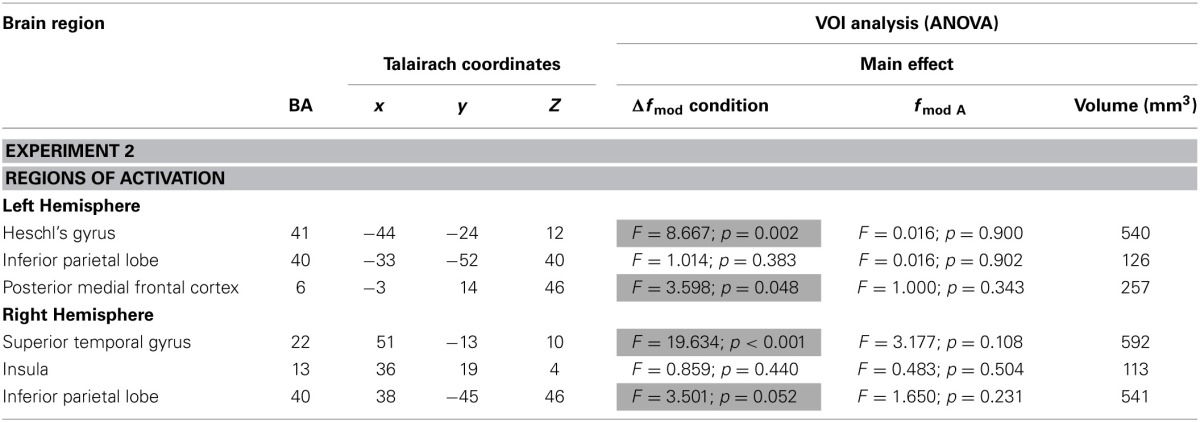
**Brain regions (BA-Brodmann area; *x,y,z*-Talairach coordinates) showing positive deflections of the BOLD signal in at least one of the three Δ*f*_mod_ conditions compared to the baseline (*t* = 4.5, *p* < 0.002) for each of the two *f_c_* (1, 4 kHz) tested in Experiment 2 and the results of ANOVAs within the resulting VOIs**.

Significant effects are highlighted in gray.

**Figure 8:**
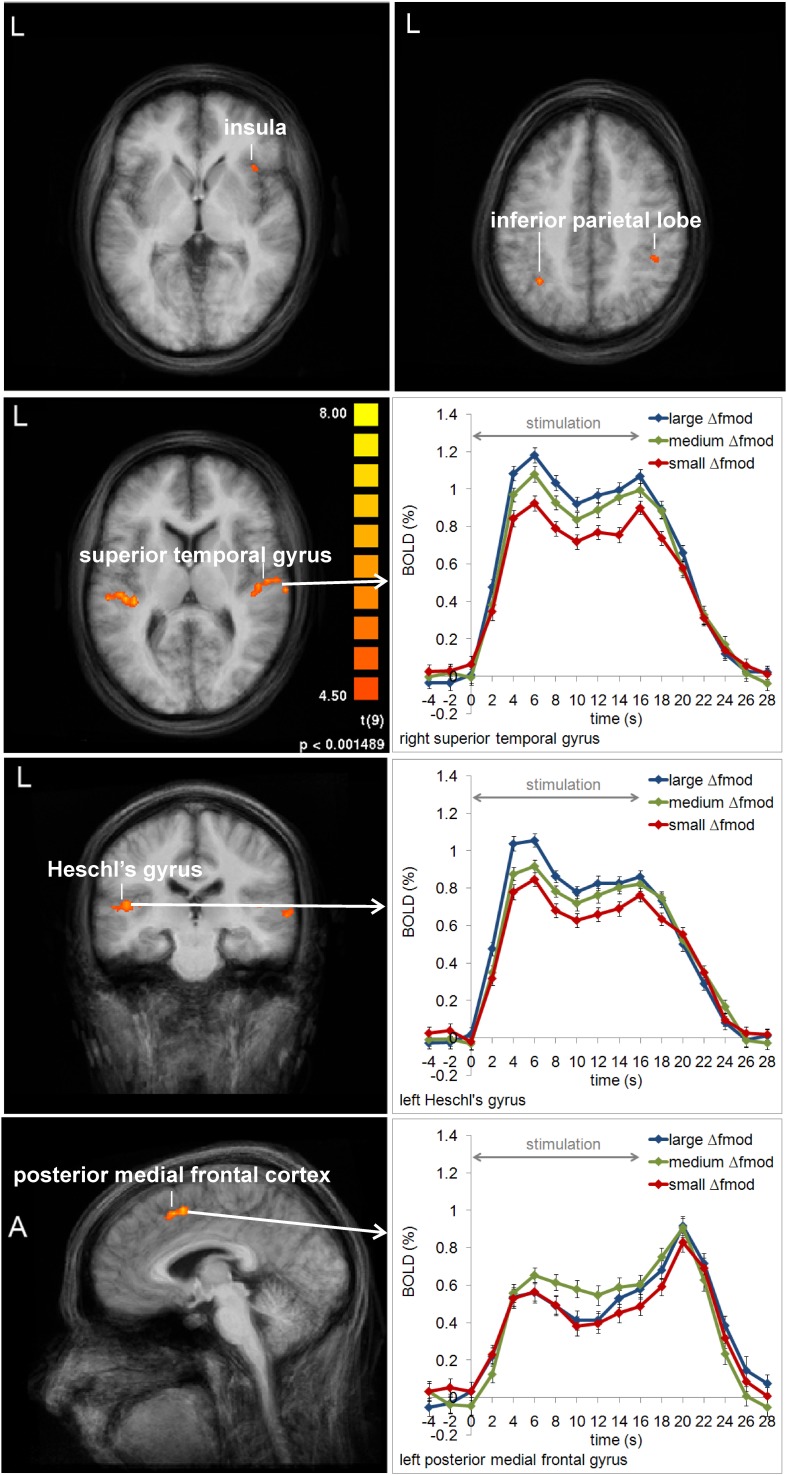
**Group average activation maps (10 subjects) and BOLD signal time courses within regions of interest in Experiment 2.** The maps depict all brain regions with positive deflections of the BOLD response in at least one of the three Δ*f*_mod_ conditions compared to the baseline (*t* = 4.5, *p* < 0.002) for each *f_c_* (1, 4 kHz). Several regions that showed significant differences between conditions are labeled and the respective averaged BOLD signal time courses are assigned. Error bars represent SEM.

In Experiment 2, a main effect of condition was found in the left HG, the right superior temporal gyrus (STG), the left MedFG, and the right inferior parietal lobe (IPL) [*F*_(2, 18)_ = 8.667, *p* = 0.002; *F*_(2, 8)_ = 19.634, *p* < 0.001; *F*_(2, 18)_ = 3.598, *p* = 0.048; *F*_(2, 18)_ = 3.501, *p* = 0.052]. In left HG and right STG the same gradual increase in BOLD response amplitude with increasing Δ*f*_mod_ was observed as in the left AC in Experiment 1 (see Figures 5, 8). *Post-hoc* tests in left HG and right STG revealed significant differences in BOLD responses between the small and the large Δ*f*_mod_ condition (*t* = 3.710, *p* = 0.005; *t* = 5.318, *p* < 0.001) and between the small and the medium Δ*f*_mod_ condition (*t* = 5.727, *p* < 0.001; *t* = 5.929, *p* < 0.001). In right STG, the large Δ*f*_mod_ condition also resulted in a significantly stronger BOLD response than the medium Δ*f*_mod_ condition (*t* = 2.698, *p* = 0.024). In left pMFC no gradual increase in BOLD response amplitude with increasing Δ*f*_mod_ was observed. In contrast, the BOLD response of the medium Δ*f*_mod_ condition was stronger than those of the small and the large Δ*f*_mod_ condition (see Figure 8) with a significant difference between the medium and the small Δ*f*_mod_ condition (*t* = 2.258, *p* = 0.050). The BOLD responses of the small and the large Δ*f*_mod_ condition were very similar (*t* = 0.643, *p* = 0.536). *Post-hoc* testing in right IPL did not reach significance. No significant main effect of the *f_c_* and no significant interaction of the factors condition and *f_c_* were found.

### Correlation between tasks and measures of stream segregation

#### Correlation between tasks

**Figure 9:**
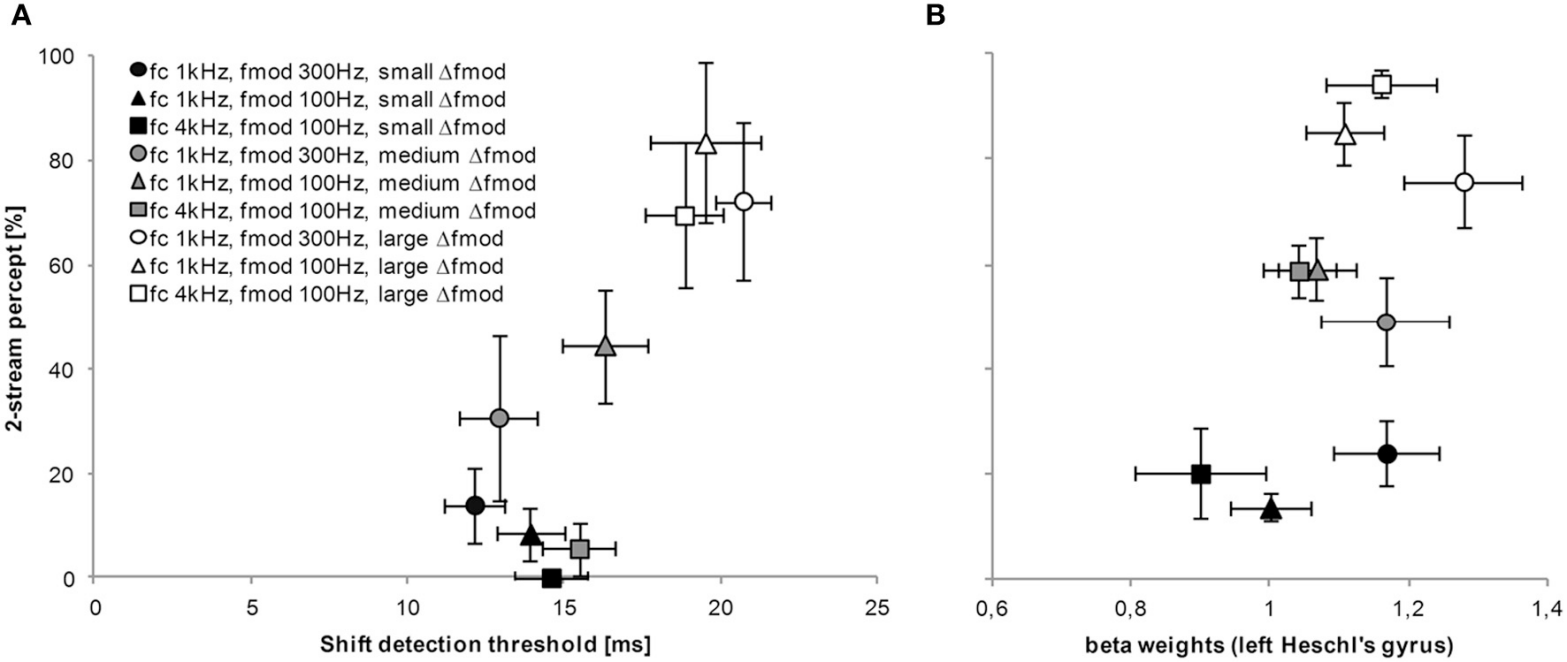
**Relationship between tasks and measures of stream segregation.** The graph in **(A)** represents the proportion of a 2-stream percept for all tested Δ*f*_mod_ stimulus conditions obtained in the subjective psychophysical task (y-axis) and the matching shift detection threshold obtained in the objective psychophysical task (x-axis). **(B)** For the purpose of comparison and as an example, the proportion of a 2-stream percept for all tested Δ*f*_mod_ stimulus conditions obtained during fMRI (y-axis) is related to the strength of BOLD responses (beta weights) in left Heschl's gyrus (x-axis). The symbols represent the three possible combinations of *f_c_*, *f*_mod A_, and Δ*f*_mod_ (see legend for values). Generally, the shading of the symbol represent the Δ*f*_mod_ stimulus condition; The darkest shading represent values for the small Δ*f*_mod_ stimulus condition, whereas the lightest shading represent values for the large Δ*f*_mod_ stimulus condition. Mean and error bars (SEM) are presented.

For all subjects and the two main experiments the mean proportion of a 2-stream percept (subjective task in quiet) and the mean shift detection threshold (objective tasks in quiet) for the tested stimulus conditions were significantly correlated (Spearman's ρ = 0.683, *p* = 0.042, Figure 9A). The Spearman's non–parametric correlation coefficients for the single subject analyses were rather large (ρ = 0.527) for all but one subjects. Only for one subject the correlation reached a significant value (*p* = 0.001).

#### Correlation between measures

For the purpose of comparison and as an example, the proportion of a 2-stream percept for all tested Δ*f*_mod_ stimulus conditions obtained during fMRI was related to the strength of BOLD responses (beta weights) in left Heschl's gyrus. Spearman's correlation of averaged group data did not reach significance (ρ = 0.450, *p* = 0.224, Figure 9B).

## Discussion

### Psychoacoustical evaluation of stream segregation by SAM

The psychoacoustical results of both experiments and both the subjective and objective task show that an increasing Δ*f*_mod_ between A and B SAM tones promotes stream segregation, being in agreement with the results of other psychoacoustical studies that evaluated stream segregation by either different SAM tones (e.g., Dolležal et al., 2012a; Szalárdy et al., 2013) or SAM noise bursts (Grimault et al., 2002).

In the present study in Experiment 1 the subjective perception of stream segregation is not affected by the *f*_mod A_ (100 and 300 Hz, respectively) of the SAM tones. Dolležal et al. (2012a), who presented sequences of SAM tones differing in multiple parameters in addition to *f*_mod A_ and *f_c_* [e.g., tone pattern (combinations of TRT and tone duration), modulation depth and presentation time] and used more steps of Δ*f*_mod_, however, observed an increasing proportion of a 2-stream percept for increasing *f*_mod A_ (30, 100, and 300 Hz). This difference between the two studies could be attributed to the differences in the range of *f*_mod A_ and Δ*f*_mod_ that was larger in the previous study by Dolležal et al. (2012a). When comparing the proportion of a 2-stream percept of the medium Δ*f*_mod_ condition, that was explicitly chosen to compare stream segregation of temporal (*f*_mod A_ = 100 Hz) vs. spectral (*f*_mod A_ = 300 Hz) cues (Table 1), spectral cues appear not to further stream segregation more than temporal cues. Next to the evaluation of the subjective streaming percept the present study also applied an objective task of stream segregation to the same stimulus conditions to be able to directly compare the streaming percept across both psychoacoustical tasks. The shift detection thresholds obtained with the objective task increased with increasing Δ*f*_mod_ between A and B SAM tones. Such an increase in the shift detection threshold with increasing feature differences between A and B signals has been observed in other studies that also presented time shifted signals in an objective task using a range of different features (frequency differences: Van Noorden, 1975; Neff et al., 1982; Cusack and Roberts, 2000; Micheyl and Oxenham, 2010; Thompson et al., 2011; differences in the starting phases of frequency components: Roberts et al., 2002; differences in fundamental frequencies: Vliegen et al., 1999). Furthermore, Divenyi and Danner (1977) also observed a sizable deterioration of the discrimination performance if the signals were made very dissimilar from each other (e.g., in frequency or intensity) even though they did not employ a paradigm that led to a streaming percept.

We also applied the shift detection task in an ABA- sequence with omitted A signals (-B---B---B--…) to determine the shift detection threshold in a condition providing no temporal reference to A signals. We observed higher shift detection thresholds in the B-only condition than for the large Δ*f*_mod_ condition of ABA- sequences with A and B signals. That difference in threshold may indicate that even in sequences with well segregated A and B signals the A signal can provide support to the detection of the time shift of the B signal. If subjects would have solely relied on the B SAM tones for their performance in both the B only condition and in the ABA- condition the thresholds should be alike.

In Experiment 2 the subjective perception of stream segregation was affected by the Δ*f*_mod_ condition and by *f_c_* (1 and 4 kHz, respectively) of the SAM tones when analyzing the subjective data from fMRI and those obtained in quiet together. The effects of the *f_c_* and Δ*f*_mod_ and their interaction was also observed by Dolležal et al. (2012a) who reasoned that the difference in the proportion of a 2-stream percept may be due to the excitation pattern differences between A and B signals being assessed by the auditory system. In the present analysis, a higher proportion of a 2-stream percept was observed for the lower *f_c_* of 1 kHz than for the higher *f_c_* of 4 kHz. At a *f_c_* of 1 kHz at least in the large Δ*f*_mod_ condition spectral cues provided for stream segregation in addition to the temporal cues that were also the prominent cue for ABA- SAM tones presented at a *f_c_* of 4 kHz. Thus, the spectral excitation pattern difference available for the lower *f_c_* of 1 kHz providing additional cues to stream segregation may be the cause for the higher amount of a 2-stream percept in that condition. The significant interaction between the condition of presentation (quiet, during fMRI) and *f_c_*, however, indicates that responses differed between both presentation conditions. The effect of *f_c_* was only prominent in quiet conditions and not in the noisy fMRI condition that may have precluded the use of excitation pattern differences. The subjective segregation percept in the noisy fMRI condition match the pattern of BOLD responses (see below). If we focus on the large Δ*f*_mod_ condition that allows comparing the amount of stream segregation elicited by spectral vs. temporal cues, we find no significant difference indicating that both type of cues have the potential to elicit the percept of well segregated streams.

In general, the proportion of a 2-stream percept was smaller for subjects that have been tested in quiet, than for subjects that have been tested in scanner noise during fMRI measurements. Especially in the medium Δ*f*_mod_ condition we observed a small amount of stream segregation that was less than expected on the basis of the previous measurements (Dolležal et al., 2012a). A similar difference in the proportion of a 2-stream percept has been observed in Experiment 1, but it did not reach significance. Wilson et al. (2007) also compared the streaming perception of subjects in quiet and during fMRI. Their results show a non-significant but higher proportion of a 2-stream percept for subjects tested during fMRI than in the quiet booth revealing a tendency that is comparable to the results of the present study. Dolležal et al. (2012a) also observed a higher proportion of a 2-stream percept in pink noise than in quiet. A general explanation for the observed effect, however, cannot be provided.

When presenting the stimulus conditions of Experiment 2 in the objective task no effect of *f_c_* on the shift detection threshold can be observed whereas an effect of Δ*f*_mod_ remained. In the subjective task the effect size of Δ*f*_mod_ was considerably larger than the effect size for *f_c_*. Since the objective task will lead to better thresholds if the subjects integrate A and B signals into a single stream (e.g., Van Noorden, 1975; Neff et al., 1982; Vliegen et al., 1999; Cusack and Roberts, 2000; Roberts et al., 2002; Micheyl and Oxenham, 2010; Thompson et al., 2011), they may be inclined to integrate more than in a subjective evaluation of the stimuli. This may reduce smaller effects of the subjective task to non-significance in the objective task.

### BOLD activity during stream segregation by SAM tones

Corresponding to the psychoacoustical results, BOLD activity in auditory cortex regions depended on the Δ*f*_mod_ between A and B SAM tones. With increasing Δ*f*_mod_ the dominant percept changed from a 1-stream to a 2-stream and the BOLD response amplitudes gradually increased. The results of Experiment 1 and 2 differ, however, in that the Δf dependent effect was observed only in left auditory cortex in Experiment 1 and in both auditory cortices in Experiment 2. Previous human imaging studies on stream segregation found either an involvement of both auditory cortices (e.g., Gutschalk et al., 2007; Wilson et al., 2007) or a specific involvement of the left auditory cortex (Deike et al., 2004, 2010). Deike et al. suggested that the involvement of the left hemisphere was caused by the specific demands on sequential analysis in the active stream segregation task. Even though the present experiments require the sequential analysis of the sound sequences, the subjects were not forced to actively group the sounds into one or the other perceptual organization but had to monitor their spontaneous perception. Therefore, one might rather suggest a stimulus driven representation of Δ*f*_mod_ in both auditory cortices and the failure to observe this in right auditory cortex in Experiment 1 might simply be explained by statistical thresholding.

The Δ*f*_mod_ dependent effect in auditory cortex regions was observed for all stimulus parameters and thus, irrespective as to whether SAM tones provide spectral, temporal or both types of cues. Several human imaging studies have described increasing neural activity throughout the auditory cortex for both differences in spectral (Deike et al., 2004, 2010; Gutschalk et al., 2005; Snyder et al., 2006; Wilson et al., 2007) and in temporal (Gutschalk et al., 2007) properties between A and B signals in streaming sequences. Hence, our finding of increasing BOLD response amplitudes in auditory cortex regions with increasing Δ*f*_mod_ between SAM tones is consistent with previous studies. Electrophysiological recording studies in animals using pure-tone paradigms suggested that frequency selectivity of tonotopically organized neurons in primary auditory cortical fields in combination with forward suppression leads to separate representations of A and B tones that contribute to the percept of two separate streams (Fishman et al., 2001, 2004; Kanwal et al., 2003; Bee and Klump, 2004, 2005; Micheyl et al., 2005). With increasing frequency separation between tones the populations of active neurons become more disjoined, leading to decreasing suppression between successive tones. It was supposed that this decrease in suppression causes the larger summed activity in auditory cortex measured using fMRI, EEG, or MEG (Gutschalk et al., 2005; Snyder et al., 2006; Wilson et al., 2007). Using harmonic tone complexes with only unresolved harmonics Gutschalk et al. (2007) suggested that suppression also accounts for the interaction of sounds with differences in temporal properties. For SAM tones Bartlett and Wang (2005) found that neurons in marmoset monkey auditory cortex show significant forward suppression of the preceding to the following SAM tone. Similarly, Itatani and Klump (2009), who used the same ABA- paradigm as in the present study and tested a large parameter space of SAM tones, observed forward suppression in multiunit responses of the auditory forebrain of awake European starlings. Related to this potential common cortical mechanism underlying stream segregation on temporal and spectral properties of sounds one may further ask the question of pitch representation at the cortex. In the present study, two stimulus conditions (Experiment 1: medium Δ*f*_mod_, Experiment 2: large Δ*f*_mod_) provided a direct comparison between spectral and temporal pitch cues on which stream segregation was based and we did not find any cortical region which showed a significant difference in BOLD responses between both cues in this comparison. This finding is consistent with the results by Hall and Plack (2009) who tested a range of pitch-evoking stimuli with different spectral, temporal, and binaural characteristics and did not find any differentiated activation within auditory cortex regions. Although differing in anatomical location, there is supporting evidence for a cue independent common pitch region in auditory cortex coming from the neurophysiological study by Bendor and Wang (2005) who found pitch-selective neurons near the anterolateral low-frequency border of the primary auditory cortex field A1 in marmoset monkeys. At the same time, the underlying mechanism for pitch coding in this region was found to depend both on the temporal and spectral characteristics of the sounds (Bendor et al., 2012).

In Experiment 1, BOLD responses in left and right Heschl's gyrus depended on the *f*_mod A_ of SAM tones with higher BOLD response amplitudes for the higher *f*_mod A_ of 300 Hz compared to the smaller one of 100 Hz. As SAM tones are characterized by three spectral peaks, i.e., the central peak representing the *f_c_* and the two sidebands (upper: *f_c_* + *f*_mod A_, lower: *f_c_* − *f*_mod A_), the stronger responses for the higher *f*_mod A_ of 300 Hz might be explained by broader spectral excitation. In addition, higher BOLD response amplitudes for the higher *f*_mod A_ were also observed in left and right insula and in the left medial part of Brodmann area 6 comprising the supplementary motor area (SMA). The involvement of these areas might be thought in the context of specific task demands other than motor processing in which both have a primary function. Specifically, the insula cortex has a role in different auditory processes, such as allocating auditory attention, temporal processing, phonological processing, and visual-auditory integration (for review, see Bamiou et al., 2003). The SMA is described as a part of the larger functional unit of posterior medial frontal cortex (pMFC) which has a function in cognitive control and particularly in performance monitoring including monitoring of response conflicts and decision uncertainty (for review, see Ridderinkhof et al., 2004). As the subjects' task was to assign their perception to one of the two perceptual alternatives, the stronger activity for the *f*_mod A_ of 300 Hz in the pMFC might reflect the monitoring of a response conflict or uncertainty in perceptual decision. In the same way, Tregellas et al. (2006) observed in the pMFC and the insular/opercular cortex an increase in BOLD activity in a “difficult” compared to an “easy” auditory temporal processing task. In their study, the subjects had to discriminate the duration of the second tone within pairs of tones and the task difficulty was adapted by varying duration differences between tones. Increasing BOLD activity in the anterior insula bilaterally with increasing task demands were also observed in a pitch discrimination and a n-back pitch memory task (Rinne et al., 2009). In the present study the task required sequence processing and rhythmic pattern perception, namely comparing the galloping ABA- rhythm (1-stream percept) to the two different isochronous rhythms (A-A-A-… and -B---B---…; 2-stream percept). Although the proportion of 2-stream perception is very similar across conditions between both *f*_mod A_ variants one might suppose that the perceptual decision might be more difficult for the higher *f*_mod A_ of 300 Hz because of specific sound qualities (e.g., timbre) other than pitch. Corresponding to this, one might suggest that the stronger BOLD response in auditory cortex for the higher *f*_mod A_ of 300 Hz also reflects the task difficulty. This notion finds support in human imaging studies providing evidences that even in sensory areas the activation can be modulated by task difficulty (Gerlach et al., 1999; Brechmann and Scheich, 2005; Reiterer et al., 2005; Harinen and Rinne, 2013).

In both experiments, cortical regions outside the auditory cortex were found which showed specific activity for medium Δ*f*_mod_ between SAM tones compared to the small and the large Δ*f*_mod_'s. In particular, the left pMFC (Experiment 2) and the left posterior cingulate gyrus (PCG) (Experiment 1) showed stronger positive and negative deflections of the BOLD signal, respectively, for the medium Δ*f*_mod_ and very similar smaller BOLD responses for the two other conditions. This activation pattern is very different from the gradual increase in activation with increasing Δ*f*_mod_ that was observed in auditory cortex regions. Whereas the activation gradient in auditory cortex rather reflects the physical differences between conditions, the BOLD responses in the pMFC and the PCG might rather be related to perceptional decision. As already mentioned above, the pMFC has a cognitive function in response conflicts and decision uncertainty. This is particularly the case in the ambiguous perceptual region where both perceptual alternatives are possible and compete with each other. Thus, the stronger BOLD response for ambiguous sequences in the pMFC might be explained by response conflicts and/or decision uncertainty. Similarly, response conflicts or decision uncertainty are equivalent to imposing higher task demands that might explain the stronger deactivation for ambiguous sequences in the PCG which is a part of the “task negative” default mode network showing decreasing activity with increasing task demands (Raichle et al., 2001; Corbetta and Shulman, 2002; Fox et al., 2005; Dosenbach et al., 2007).

Our fMRI results can be summarized as follows. In auditory cortex stream segregation on SAM tones showed the same Δf dependent BOLD responses as other streaming stimuli. In contrast, BOLD activity in regions outside the auditory cortex rather appear to reflect the perceptual decision and specifically the higher task demands caused by specific stimulus characteristics or by perceptual ambiguity leading to response conflicts and decision uncertainty, respectively. The involved regions differ from those observed in other studies and we did not find significant activation in any of the regions reported in Cusack (2005) (intraparietal sulcus), Kondo and Kashino (2009) (Thalamus), and Dykstra et al. (2011) (e.g., middle temporal and frontal gyri). This might be explained by general differences in the approaches: Cusack (2005) and Kondo and Kashino (2009) examined ambiguous streaming sequences to find correlates of different perceptual organizations and perceptual switches, respectively, whereas the present study examined stream segregation across the domains of perceptual dominance and ambiguity by varying the stimulus parameters. The study by Dykstra et al. (2011) also compared different Δf conditions and found that the middle temporal and frontal gyri showed the same increase in neural activity with increasing Δf as the auditory cortex. They, however, did not observe a specific response for ambiguous stimuli. This discrepancy must be resolved in future studies.

### Comparison across tasks and measures

A direct comparison across psychoacoustical tasks of stream segregation showed a correlation across all subjects and experiments (Figure 9A). The results of the objective task mirror the results obtained by the subjective task, thus the shift detection threshold as well as the proportion of a 2-stream percept increased with increasing Δ*f*_mod_ between A and B SAM tones. Such a correlation reveals that both the proportion of a 2-stream percept (subjective task) as well as the shift detection threshold (objective task) can represent the amount of stream segregation. These results are in agreement with a study by Micheyl and Oxenham (2010) who presented pure tones in ABA- sequences with frequency differences between A and B tones and also correlated the proportion of a 2-stream percept with the shift detection experiment. A comparison across measures (subjective streaming percept and BOLD responses (beta weights) in left Heschl's gyrus) did not show a significant correlation even though a relatively high Spearman's rho was observed for the mean values of both measures (Figure 9B). In the exemplary figure of the correlation of humans perception and BOLD responses in left Heschl's gyrus a trend similar to the one observed in the figure of the correlation across psychoacoustical tasks (see Figure 9A) can be observed, showing an increasing proportion of a 2-stream percept with increasing beta weights measured in BOLD responses.

### Conflict of interest statement

The authors declare that the research was conducted in the absence of any commercial or financial relationships that could be construed as a potential conflict of interest.
